# Mutants on the Small Screen

**DOI:** 10.1371/journal.pbio.0020262

**Published:** 2004-08-17

**Authors:** Jonathan Hodgkin

## Abstract

A recent television series in the UK celebrates the genetic diversity of human life

My graduate adviser, Sydney Brenner, used to exclaim ‘Revenons à nos mutants!’ as he sat down at the bench to search for yet more genetic variants of C. elegans. Those mutants won him a Nobel Prize, some thirty years later. Armand Leroi is another aficionado of C. elegans mutants, but he decided to write a book—and then to make a television series—on mutants of humanity, not of worms. He says at the beginning and end of the series: “We are all mutants, but some of us are more mutant than others.” This is a good slogan, and very proper for embracing humanity as a whole. He backs it up with an aphorism from Etienne Geoffroy Saint-Hilaire, pioneer of teratology, who proclaimed: “There are no monsters and Nature is one.” What Geoffroy meant was that abnormalities provide clues to normal processes, and hence are invaluable to science, if they can be properly understood. But monsters and mutants are, undeniably, fascinating in their own right.[Fig pbio-0020262-g001]

**Figure pbio-0020262-g001:**
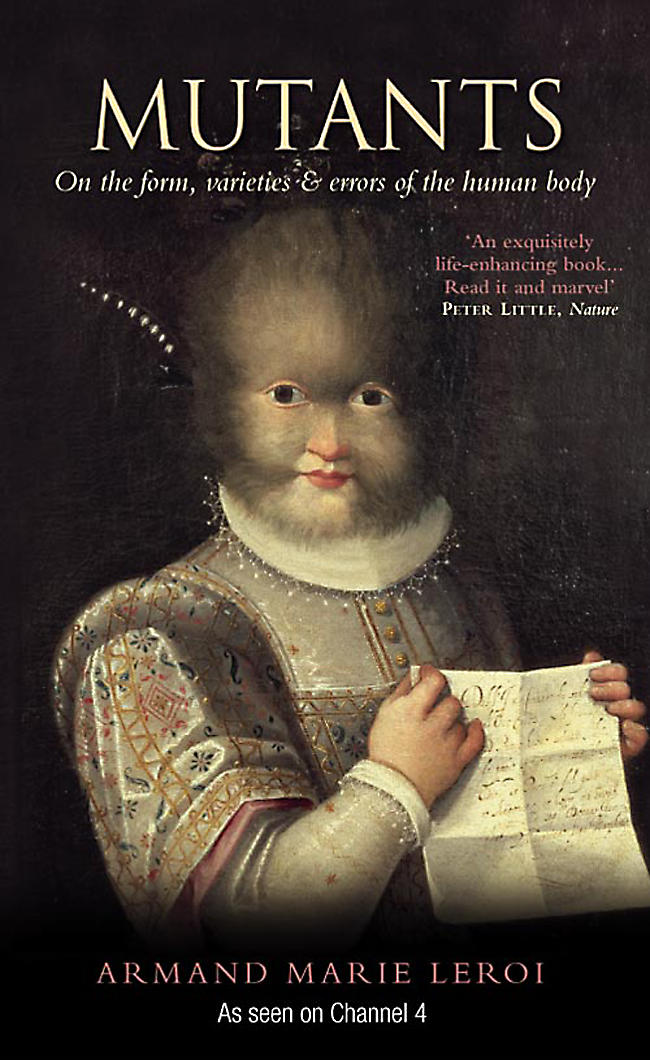


*Mutants*, the book, is excellent: impressively researched and illustrated and extremely well written. The resulting television series on Channel 4 in the United Kingdom has a distinctly different impact. The series covers only a few of the subjects dealt with in the book, and handles the material in a different way. Inevitably, television can't include much science or scholarly detail, but it compensates with human images that are wholly gripping—both the preserved specimens and the living subjects who talk about the strange conditions that they live with. It's a freak show, but a freak show with thoughtful scientific commentary.

Each of the three programmes in the series has a particular theme. The first, ‘The Mystery of Growth’, is devoted mainly to skeletal disorders. We meet Carole Ozel, who copes with extraordinary courage with a terrible disease called fibrodysplasia ossificans progressiva (FOP), in which bony tissue forms throughout the body, gradually immobilizing the body in a second skeleton. Later we encounter a crew of charming and articulate dwarfs taking time out from a disco at the Reno convention of the Little People of America. They are happy to be called dwarfs or little people, but midget is no longer an acceptable term. Being a dwarf, in fact, is sometimes a ticket to fame and fortune, as in the case of Joseph Boruwlawski, last of the court dwarfs, who enchanted European royalty, married a beautiful woman, and lived happily to the age of 98.

The second part, ‘The Dangerous Womb’, is about birth defects, conjoined twins, and basic embryology. The makers of the piece went to the trouble of getting the developmental biologist Eddie de Robertis to reconstruct the classic experiment of Mangold and Spemann that revealed the underlying basis of some of these defects. The programme goes into some detail about what is now known about the molecular basis of normal and abnormal development, and how we can begin to explain such extraordinary forms as that of the Parodi twins, who had distinct heads and shoulders, but merged into a single torso and a single pair of legs.

The third programme, ‘The Meaning of Beauty’, deals with lesser but still striking abnormalities such as albinism and hypertrichosis (excess hair). It also moves into contentious areas, in a frank discussion of race genetics. Leroi makes the essential point loudly and clearly: there is much more genetic variation within any village on earth than there is between different human populations. We are extraordinarily unified, from a genetic standpoint. But, as he notes, the idea of race persists, and about 7% of global genetic variation in human DNA includes AIMs, ancestry informative markers. Some of these distinguish, for example, Africans and Europeans and provide objective information about the ancestry of different human populations.

This being television, the series closes with a discussion of beauty, which Leroi proposes to be simply the absence of visible mutant defects, using Saira Mohan, *Newsweek*'s idea of human facial perfection, as an exemplar. Yes, she looks nice, but nice can be boring. Beauty, above all other human attributes, is profoundly influenced by culture, and it is hard to take this interpretation of beauty as an adequate explanation, rather than just a pretty way to finish the series.

For the most part, Leroi makes an agreeable and humane commentator, though he is not immune to the slight self-satisfaction that seems to overcome all scientists on television. The camera also spends an excessive amount of time dwelling on him, to a point where it becomes irritating to see him walking—frequently in slow motion— into yet another museum or laboratory. Sometimes the focus on the presenter pays off, as when we see the six-foot scientist looking like a small child beside Chris Greener, the tallest man in Britain, or witness Leroi's faint chagrin at discovering that his DNA is mostly European, despite his cosmopolitan family history. More questionable are the bits where he paddles casually through a tub of preserved viscera from some long-gone sufferer from situs inversus (mirror-reversed organs) and succumbs to laughter at the sight of Ditto, the amazing two-faced pig. This may be an honest attempt at portraying the conflicted reactions we all have to abnormality, but it seems bound to cause trouble.

Arty camerawork is a running feature of the programmes, and there is a great deal of smoke and mirrors about the whole production. Perhaps this is deliberate, reminding us that it is hard to look directly at extreme deformity, but there is an air of ‘I wants to make your flesh creep’ about the many sidelong shots of mutant babies in bottles in the Vrolik Museum, to which we return again and again throughout the series. Viewing the already distorted fetuses through further distorting camera angles and under green lighting doesn't really achieve anything. The treatment begins to resemble the first *Alien* movie, in which the audience was never allowed to see the monster directly.

In the end, what is most memorable about these programmes is the living people themselves, and how they have coped with their various genetic disorders. It is very touching to see the home movies of Tiffany York, born with mermaid syndrome, or sirenomelia (in which the legs are fused together), taking her first tottery steps after corrective surgery, and to listen to her talking philosophically about her life as she floats in a Florida swimming pool. It is similarly cheering to hear from the dwarfs and albinos, or from Chuy Aceves, who has hypertrichosis and looks like the original Hollywood wolfman but suffers no ill effects and is proud of his rare condition. For these sections alone, let alone the serious and well-explained scientific background, the series is well worth seeing. It makes one feel surprisingly good about the human race and the human spirit.

